# Combined Korean medicine therapies in children with allergic rhinitis: A multi-center, observational explanatory registry trial: A study protocol

**DOI:** 10.1097/MD.0000000000028181

**Published:** 2021-12-23

**Authors:** Hongmin Chu, Bo-Hyoung Jang, Eunkoung Lee, Seunghwan Moon

**Affiliations:** a Daecheong Public Health Subcenter, Daecheong-myeon, Ongjin-gun, Incheon, Republic of Korea; b Department of Preventive Medicine, College of Korean Medicine, Kyung Hee University, Seoul, Republic of Korea; c Ham-soa Korean Medicine Clinics, Seoul, Republic of Korea.

**Keywords:** acupuncture, allergic rhinitis, complementary and alternative medicine, Korean medicine treatment, registry study

## Abstract

**Introduction:**

Allergic rhinitis (AR) is the third most prevalent disease in early and middle adolescence in South Korea and one of the most common allergic diseases worldwide. Due to the ineffectiveness and frequent side effects of conventional medications for AR (such as antihistamines, corticosteroids) complementary and alternative medical (CAM) therapies have been in the spotlight. Although previous clinical trials conducted on AR with CAM showed efficacy and safety, these research results have limitations in that they did not estimate the effectiveness of combining multiple interventions. In this respect, this study aims to investigate the efficacy and safety of combined Korean medicine therapy by establishing an observational registry study at 13 Korean medical clinics that specialize in treating pediatric rhinitis.

**Methods:**

This is a prospective, observational, registry study of adolescent patients with AR. After screening, eligible subjects will be allocated to the registry. The patients will undergo a 4-week treatment and a 4-week post-treatment follow-up. The primary outcome will be the change in the average total nasal symptom score evaluated from baseline to the end of treatment. The secondary outcomes will include the numerical range scale, quality of life questionnaire in Korean children with AR, and the Pediatric Allergic Disease Quality of Life Questionnaire. KiFDA 3.0 will be measured for explanatory application on adolescents. Medical cost data and characteristics of patients such as weight, height, and sex will be collected by researchers.

**Discussion:**

This is the first multi-center observational registry study to compare combined Korean medicine treatment for AR. The results of this study will shed light on the effectiveness and safety of Korean medicine treatments for the treatment of patients with AR.

**Trial registration:**

KCT0006625 (2021.09.30)/IRB approval: Kyung-hee University Institutional Review Board (approval number: KHSIRB-21-358-1 [NA]).

Trial Status: Protocol version 1.2(2021.09.16).

## 1 Introduction

Allergic rhinitis (AR) is the third most prevalent disease in early and middle adolescence, and almost 940,000 children receive treatment in South Korea per year. Nevertheless, allergic rhinitis (AR) shows a 3% increase rate every year due to living in an indoor environment as well as westernized eating and living habits. Previous research estimated that ∼2% to 25% of the global population is affected by AR.^[1–3]^ Due to the high prevalence and high cost of allergic rhinitis, a substantial economic burden on patients and healthcare systems has been reported.^[4–6]^

Typical symptoms of AR include rhinorrhea, sneezing, coughing, itching in the eyes or nasal region, and nasal obstruction.^[7]^ Since these symptoms greatly reduce the quality of life, AR patients are often prone to problems such as drug dependence or steroid overdose.^[8]^ Conventional therapies for AR include antihistamines, intranasal corticosteroids, and surgical treatments, which can lead to repeated adverse events. In this regard, the application of complementary and alternative medicine (CAM) has been in the spotlight.^[9,10]^ Various trials on the treatment of AR with CAM interventions such as acupuncture, herbal medicine, acupressure, and herbal patching have shown efficacy and safety. However, there is insufficient research that investigates the effectiveness of combined CAM interventions in AR treatment.^[11–21]^

In the outpatient department of the Korean Medicine clinic, multiple interventions are involved in the treatment procedure, whereas existing studies do not adequately address this pragmatic approach.

To overcome these limitations in previous research, we established an observational registry study on 13 Korean medicine clinics that specialize in treating pediatric rhinitis. Through this study, we expect to examine the efficacy and safety of AR through a combined oriental medicine treatment method.

## 2 Methods/design

### 2.1 Study registration

This study was registered with the Clinical Research Information Service (CRIS) of the Korea National Institute of Health (NIH), Republic of Korea (KCT0006625).

### 2.2 Study design

The aim of the proposed study is to conduct a multi-center, observational registry study with 120 children with physician-diagnosed AR to assess whether Korean medicine treatment might be effective at improving AR symptoms as the primary outcome. As an explanatory analysis, the study will collect patient‘s Korean medicine pattern diagnosis data and assess whether the KiFDA 3.0 questionnaire is an effective tool for classifying AR‘s symptoms in children. It is further expected that the safety of Korean medicine therapy can be estimated through this study.

**Figure 1:**
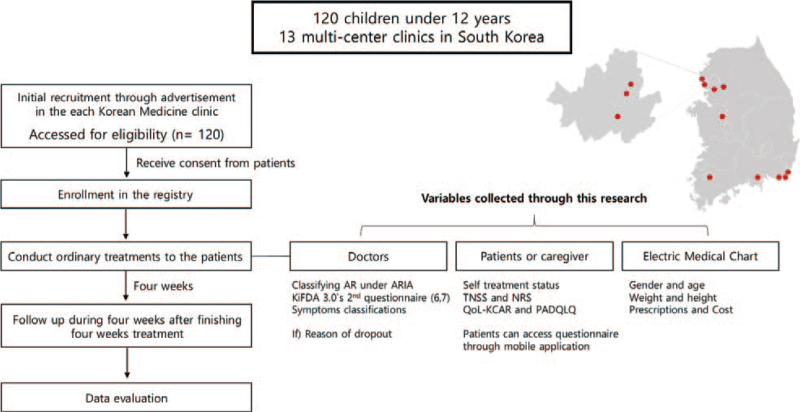
Clinical trial design. Target enrollment of 120 children will be accomplished by advertisements posted in 13 medical clinics specializing in Korean medicine (CAM). Subjects providing informed consent and meeting the study's inclusion/exclusion criteria will be enrolled in the registry. A 4-week intervention period will be followed by a 4-week follow-up period. Data collected will then we analyzed. Data will be collected through three sources: Clinic doctors, Patients and/or their caregivers and electronic medical chart (records).

Subjects or their caregivers who voluntarily sign a written informed consent form after receiving a sufficient explanation of the benefits and risks of participating in the study will be evaluated for compliance with the inclusion and exclusion criteria through a screening process on their first visit. After screening, eligible subjects will be assigned to the registry. The patients will undergo 4 weeks of treatment with combined Korean medicine treatment (cKMT) which consists of acupuncture, herbal medicine, electro-moxibustion, cupping therapy, laser therapy and ointment. To reflect the primary care clinics and outpatient department environment as much as possible, doctors who treat participants will select the type of intervention after evaluating the child's condition. After finishing the 4-week treatment, a 4-week follow-up online questionnaire will be administered. The detailed study procedure is shown in Figure 1.

The study protocol was approved by the Institutional Review Board (IRB) of Kyung-hee University (approval number KHSIRB-21-358-1(NA)), and informed consent will be obtained from each participant.

### 2.3 Participants

This registry study will be conducted on children under 12 years of age. In this respect, caregiver's of subjects can sign a written consent form after receiving a sufficient explanation. The consent and explanation for children will be prepared, and consent will be obtained from the children.

#### 2.3.1 Setting

**Figure 2:**
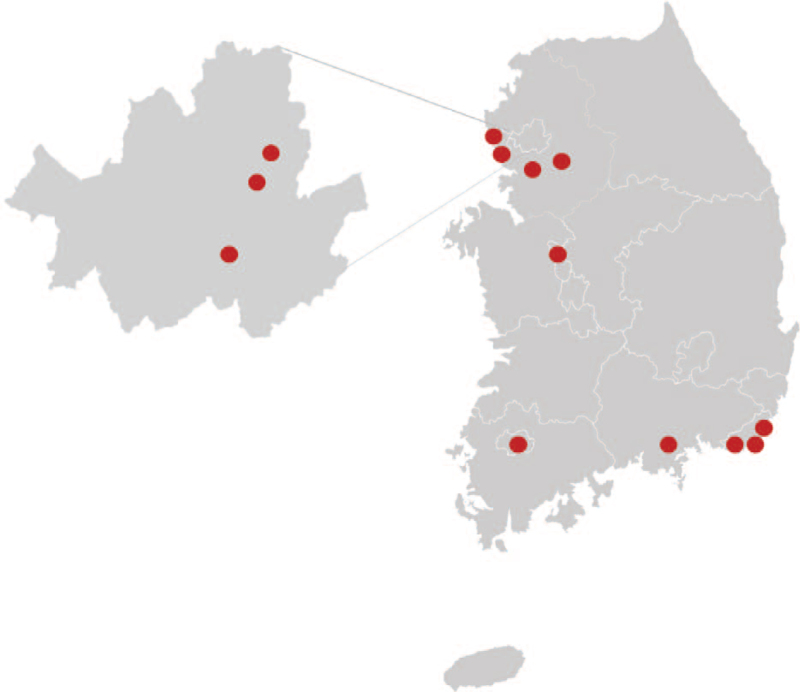
Locations of Korean medicine clinics. A total of 13 Korean medicine (CAM) clinics located throughout South Korea will participate in the study.

This study will be conducted in 13 Ham-soa Korean medicine clinics located in the Republic of Korea. All subjects will be recruited from the daily outpatient department of Korean medicine clinics that are participating in this study. The locations of the Korean medicine clinics are shown in Figure 2.

#### 2.3.2 Inclusion criteria

- Children under 12 years of age who visited the Ham-soa Korean Medicine Clinic for AR treatment.

Diagnosis of AR with the Allergic Rhinitis and its Impact on Asthma (ARIA) criteria

Intelligible yes/no response

- The ability to understand basic instructions in spoken Korean.
- Availability to participate in the investigation for the duration of the 8-week study.
- Accessibility to mobile questionnaires.
- Children or caregivers who agreed to participate and signed the consent form after hearing a clear explanation of the purpose and characteristics of this clinical study.

#### 2.3.3 Exclusion criteria

- Those who participated in other clinical studies
- Diagnosed with allergic rhinitis but has a history of visiting another medical institution and receiving treatment for AR within the previous 3 months
- Those who refuse to participate in clinical research or provide consent
- Those judged to be inadequate for clinical research or have any other reason deemed inadequate to participate in research by the medical staff.

#### 2.3.4 Early termination and dropout criteria

- Participants or caregivers request to quit the examination or want to drop out from the study.
- Misalignment with inclusion or exclusion criteria.
- If the principal investigator judges that the study progress is inadequate or there is a serious violation (adverse event, patient confidentiality, etc) during the research study.

#### 2.3.5 Plan for recruitment of study participants

Patients who visit the Korean Medicine Clinic's outpatient department for treating AR will become candidates for patient group recruitment. Advertisements on websites or newspapers will also be used.

#### 2.3.6 Registration of participants

Patients with AR who meet the inclusion and exclusion criteria will be assigned to the registry. After enrolling in the registry, participants‘ medical information related to AR will be collected during their medical treatment.

### 2.4 Intervention

Ham-soa Korean medicine clinics’ treatments protocol for treatment of AR is as follows, and participants will receive treatments according to this protocol. Changes in treatments judged as necessary by doctors will be recorded separately.

#### 2.4.1 Herbal medicine

Herbal medicine such as Socheongryong-tang, Okbyeongpung-san, Bojungikgi-tang, Hyeonggaeyeonkyo-tang, and Samsoeum are used in the treatment. If addition and subtraction of medical herbs from the original formula is performed by doctors, the kinds and doses of the herbs will be recorded. Extract or powder types of herbal medicine can also be used in the treatment based on the doctors’ judgment.

#### 2.4.2 Acupuncture and moxibustion

Acupuncture treatment will be performed by Korean medicine doctors. Additional information such as needle type, acupoint name, depth insertion, and duration times will be charted. Furthermore, electronic moxibustion can be used in treating the study participants.

#### 2.4.3 Cupping therapy

Cupping therapy can used on the Back-shu point. The attachment regions and duration times will be charted.

#### 2.4.4 Laser therapy or infrared therapy

If infrared therapy or laser therapy is used in the treatment, doctors will indicate the type of treatment.

#### 2.4.5 Ointment and other interventions

If ointment or other interventions are used in the treatment, doctors will indicate the type of treatment.

### 2.5 Outcomes

First, doctors will diagnose allergic rhinitis under the ARIA guidelines and classify the patients according to the criteria. After being allocated to this registry, doctors will conduct the total nasal symptom score (TNSS) questionnaire. TNSS will be the primary endpoint of this study.^[22]^ TNSS will be recorded as a weekly average from baseline to the end of the trial. The secondary endpoints will be the Numeric Rating Scale (NRS), Quality of Life Questionnaire in Korean children with allergic rhinitis (QoL-KCAR), and the Pediatric Allergic Disease Quality of Life Questionnaire (PADQLQ).^[23,24]^ Participants‘ characteristics, such as height, weight, and gender, will also be collected by the researchers. For precise outcome assessment, researchers will be specifically trained and familiar with each type of examination.

Meanwhile, exploratory factors such as KiFDA 3.0, will be assessed. KiFDA 3.0 consists of two questionnaires. If a score is obtained between 11 and 21 in the 1st questionnaire, the 2nd questionnaire is not completed, but if 10 or fewer points are obtained, the 2nd questionnaire is conducted.^[25]^ The 1st questionnaire consists of seven symptoms (facial color, digestive problems, gas, stool condition, general condition, appetite, and body mass index using a four-point Likert scale from 0 to 3 [0 = no symptoms, 1 = Occasionally, 2 = Frequently, 3 = Always]). The 2nd questionnaire also consists of seven questions (coldness, drinking water, general facial color, detailed stool condition, urine color, rhinorrhea's viscosity and color, and nasal endoscopy status. Questions 1 to 5 will be answered by the patient, and 6 and 7 will be evaluated by the doctors.

### 2.6 Sample size and statistical analysis

Since this is an observational registry study, a sample size calculation is not required. However, because 13 Ham-soa Korean medicine clinics will be involved in this study, researchers plan to set the number of participants at a minimum of 120. Statistical analysis will be conducted using the Statistical Package for the Social Sciences (SPSS) for Windows, version 20.0. Continuous variables will be expressed as means (standard deviations) or medians (quartiles), and categorical variables will be expressed as numbers and percentages of patients. The study will compare clinical outcomes between the baseline and termination.

### 2.7 Data collecting, processing, and monitoring

All collected data will be managed using a confidential online database. Files containing information from the participants will be stored in a locked filing cabinet at Kyung-hee University. As this study is a registry study, no further monitoring will be needed.

### 2.8 Ethics and dissemination

#### 2.8.1 Ethical approval

This study was approved by the IRB of Kyung-hee University, Seoul, Republic of Korea (KHSIRB-21-358). The study will be conducted in accordance with the Declaration of Helsinki.

#### 2.8.2 Consent statement and obtaining written consent

Participants will receive consent statements and voluntarily sign after listening to a full description of the study's purpose, possible adverse events, and safety. Signed consent will be obtained from all the participants’ parents or guardians on paper. Verbal consent will be obtained from the children. If a participant or their legal representative cannot read or understand the consent, the researchers will provide a manual for children and explain the full procedure for them.

#### 2.8.3 Compensation for severe or emergency adverse events

As this study is an observational registry study, doctors will conduct conventional treatments that are currently administered routinely at the Ham-soa Korean Medicine Clinic. In this respect, there is little harm anticipated, other than immediate side effects. However, in case of emergency or severe side effects, a compensation award will be prepared separately, and the study participant will be provided with the best possible treatments. Naturally, participants can withdraw their enrollment whenever they want.

### 2.9 Adverse events

Any minor or major adverse event associated with the study will be noted and reported by the researchers. Any unintended effects will be detected and recorded.

### 2.10 Changes to study protocol

Any changes to the protocol that may affect the performance of the study, its benefits, or the safety of the subjects or changes, including the study design, sample size, and study procedures, will be approved by the IRB committee before implementation.

## 3 Discussion

This study aims to establish an observational study registry and collect clinical data from 13 multi-center Korean medicine clinics that specialize in treating pediatric rhinitis. The protocol of this study was developed according to the Strengthening the Reporting of Observational Studies in Epidemiology (r) statement and Standard Protocol Items: Recommendations for Interventional Trials (SPIRIT) statement.^[26,27]^

This protocol describes a combined Korean medicine therapy that we propose will have a positive effect not only on symptom improvement and daily functioning of children with AR, but also on other measures such as quality of life and cost-effectiveness. This protocol has four strengths. First, it will be the first registry study of AR with combined Korean medicine therapies and more than 10 clinics are involved in this observational registry. Second, to reflect a real-life clinic environment that utilizes multiple treatments rather than just a single intervention, various interventions such as laser therapy and ointment data will be included. Third, to increase the response rate and for the convenience of participants, a mobile application has been developed for the questionnaire that can be answered through a smartphone device and data collected electronically. Fourth, the results of this study are expected to provide consolidated evidence for the effectiveness and safety of Korean medicine treatments for the treatment of patients with AR.

## Acknowledgments

This study was supported by a grant of the project, Guideline Center for Korean Medicine, National Institute for Korean Medicine Development (HI16C0275).

## Author contributions

HC: Investigation, Methodology and writing-original draft

BJ: Conceptualization, Data curation, Investigation and writing-reviewing & editing

EL: Data curation and writing-reviewing & editing

SM: Data curation, Investigation and writing-reviewing & editing

Ham-soa Clinics KM doctors group: Data curation

**Conceptualization:** Bo-Hyoung Jang.

**Data curation:** Bo-Hyoung Jang, Eunkyuon Lee, Seunghwan Moon, Hamsoa KM Doctors group.

**Investigation:** Hongmin Chu, Bo-Hyoung Jang, Seunghwan Moon.

**Methodology:** Hongmin Chu.

**Writing – original draft:** Hongmin Chu.

**Writing – review & editing:** Bo-Hyoung Jang, Eunkyuon Lee, Seunghwan Moon.
